# Assessing validity evidence for a serious game dedicated to patient clinical deterioration and communication

**DOI:** 10.1186/s41077-020-00123-3

**Published:** 2020-05-27

**Authors:** Antonia Blanié, Michel-Ange Amorim, Arnaud Meffert, Corinne Perrot, Lydie Dondelli, Dan Benhamou

**Affiliations:** 1 Centre de simulation LabForSIMS, Faculté de médecine Paris Saclay, 94275 Le Kremlin Bicêtre, France; 2 Département d’Anesthésie-Réanimation chirurgicale, CHU Bicêtre, 94275 Le Kremlin Bicêtre, France; 3 CIAMS, Université Paris-Saclay, 91405 Orsay Cedex, France; 4 CIAMS, Université d’Orléans, 45067 Orléans, France; 5 IFSI Sud Francilien, 91106 Corbeil-Essonnes, France

**Keywords:** Serious game, Simulation, Validity evidence, Patient deterioration

## Abstract

**Background:**

A serious game (SG) is a useful tool for nurse training. The objectives of this study were to assess validity evidence of a new SG designed to improve nurses’ ability to detect patient clinical deterioration.

**Methods:**

The SG (*LabForGames Warning)* was developed through interaction between clinical and pedagogical experts and one developer. For the game study, consenting nurses were divided into three groups: nursing students (pre-graduate) (group S), recently graduated nurses (graduated < 2 years before the study) (group R) and expert nurses (graduated > 4 years before the study and working in an ICU) (group E). Each volunteer played three cases of the game (haemorrhage, brain trauma and obstructed intestinal tract). The validity evidence was assessed following Messick’s framework: content, response process (questionnaire, observational analysis), internal structure, relations to other variables (by scoring each case and measuring playing time) and consequences (a posteriori analysis).

**Results:**

The content validity was supported by the game design produced by clinical, pedagogical and interprofessional experts in accordance with the French nurse training curriculum, literature review and pilot testing. Seventy-one nurses participated in the study: S (*n* = 25), R (*n* = 25) and E (*n* = 21). The content validity in all three cases was highly valued by group E. The response process evidence was supported by good security control. There was no significant difference in the three groups’ high rating of the game’s realism, satisfaction and educational value. All participants stated that their knowledge of the different steps of the clinical reasoning process had improved. Regarding the internal structure, the factor analysis showed a common source of variance between the steps of the clinical reasoning process and communication or the situational awareness errors made predominantly by students. No statistical difference was observed between groups regarding scores and playing time. A posteriori analysis of the results of final examinations assessing study-related topics found no significant difference between group S participants and students who did not participate in the study.

**Conclusion:**

While it appears that this SG cannot be used for summative assessment (score validity undemonstrated), it is positively valued as an educational tool.

**Trial registration:**

ClinicalTrials.gov ID: NCT03092440

## Background

Detection of patient deterioration is a major healthcare problem since a modification of physiological parameters often precedes acute patient clinical deterioration by 6 to 24 h [1–3]*.* The association of (i) early detection, (ii) speed of response and (iii) quality of clinical response influences the patient prognosis. Many studies have shown that delayed diagnosis of an ongoing complication increases morbidity and mortality [3]. The education of nurses, who are frontline healthcare providers, is therefore essential.

When nurses are confronted with a case of clinical deterioration, they must not only recognise the incident but also notify the medical team. The use of a safe and standardised communication method such as the SBAR method [4, 5] improves patient safety [6, 7]. Training in understanding the role of appropriate communication and in the use of such a tool is therefore essential for healthcare professionals.

When compared with high-fidelity simulation, serious games (SG) possess an interesting immersive capacity and offer the advantage of training a large number of healthcare professionals in a limited amount of time using reduced educational resources [8, 9]. Moreover, SG are standardised cases providing automated feedback. SG can be used to develop both technical and non-technical skills [10–12]. We developed a SG called *LabforGames Warning,* which aims to improve nursing students’ interprofessional communication behaviour and their ability to detect patient clinical deterioration. Training in these essential skills will be soon added to the French nursing curriculum. In another study by our team, clinical reasoning was assessed in nursing students after a training course dedicated to the detection of patient deterioration, comparing a serious game-based simulation course with a traditional teaching course [13]. Although no significant educational difference was found between the two methods, participants reported greater satisfaction and motivation with serious game-based simulation training. However, the validity of this SG needed to be assessed before it could be used widely in professional healthcare education [14, 15]. The objective of this study was to assess the validity evidence of *LabForGames Warning* before the game is used in educational activities.

## Methods

### SG development

The SG project was promoted by the Paris Sud University simulation centre (LabForSIMS) in collaboration with four nursing schools (Sud Francilien, Perray Vaucluse, Paul Guiraud and Etampes) through a grant from the Ile-de-France Healthcare Regional Agency (ARS).

Three virtual clinical cases described below were developed through iterative dialogues between the pedagogical team and the developer (Interaction Healthcare®, Levallois-Perret, France). The medical instructors were clinical experts (teachers at four nursing schools and anaesthesiologists) and were also involved in the simulation centre. The educational objectives chosen for the SG were the detection of clinical deterioration and interprofessional communication. In the game, nurses are required to identify clinical deterioration in three different clinical situations and to notify the medical team accordingly based on the patient’s clinical severity. *LabForGames Warning* derived its name from the early warning scoring system described in literature [16]. As the SG focuses on nursing students, the objectives needed to conform to the French nurse training curriculum [17].

In each clinical scenario, three consecutive steps (mildly abnormal, moderate aggravation and serious condition) were constructed to reproduce a specific complication of increasing severity in order to introduce the concept of early warning signs [16]. The three cases were of equal moderate complexity. The clinical cases created were as follows:

- Case 1 (post-operative haemorrhage): an adult female patient having undergone a scheduled total hip prothesis earlier in the day and who is lying in her ward room bed immediately after arrival from the post-anaesthesia care unit. Post-operative haemorrhage from the surgical site is occurring progressively.
- Case 2 (brain trauma): an elderly patient with dementia living in a nursing home whose anticoagulation is associated with progressively developing neurological deterioration following brain trauma from a fall.
- Case 3 (obstructed intestinal tract): a schizophrenic patient hospitalised in a psychiatric ward with intestinal obstruction of progressively increasing severity.

Learning safe and standardised communication was an additional educational objective of the game [6, 7]*.* We chose to train nursing students in the SBAR method, (Situation, Background, Assessment, Recommendation), which has been translated into French by the French Health Authority [5].

**Fig. 1:**
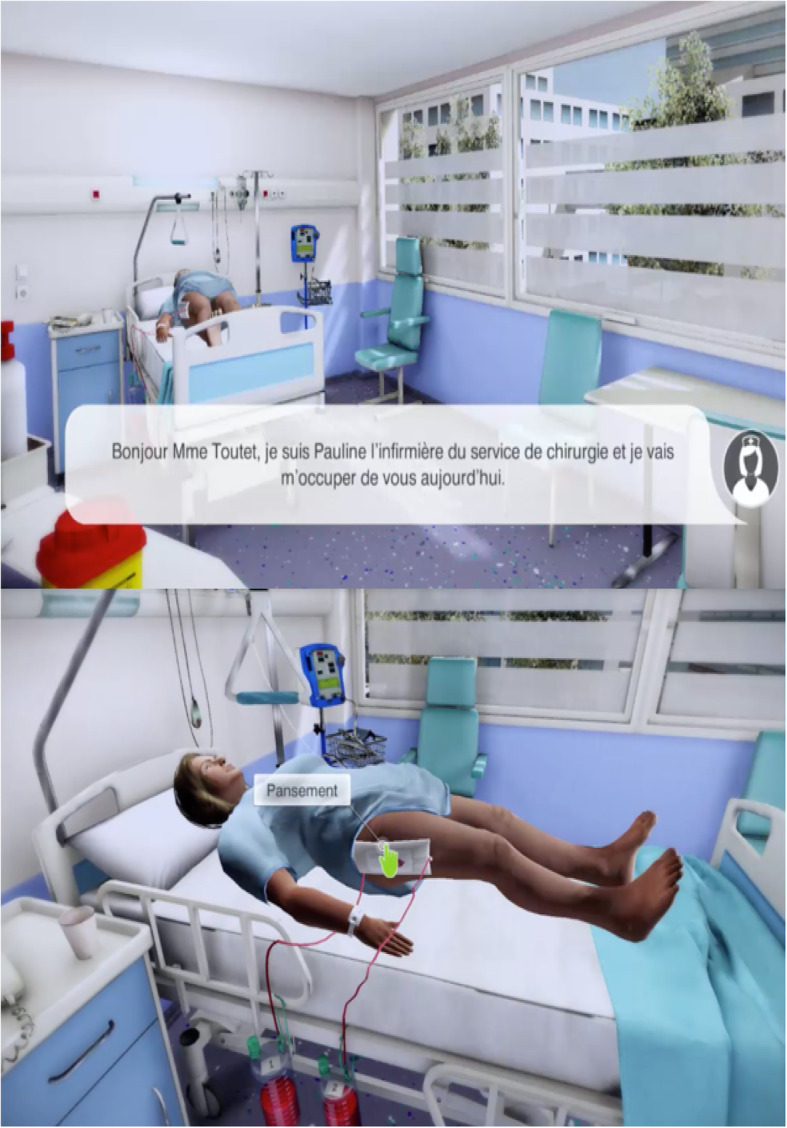
Screenshots of *LabForGames Warning*

During the case, participants can perform different actions: history taking, clinical exams (circulatory assessment, neurologic assessment, skin temperature, etc.), care report writing and calling the physician. Screenshots of *LabForGames Warning* are provided in Fig. 1 and Additional file 1: panel a-f.

At the end of each scenario, virtual automatic feedback was presented to the participant. Feedback included main guidelines and key messages about the detection of patient clinical deterioration (in general and in the specific case) and the SBAR method, as well as individualised global and detailed scoring (see Additional file 1: panel g, for an example).

The criteria for the detailed scoring had previously been established by the pedagogical team. The participant’s clinical examination actions (checking arterial pressure, pain, etc.) and his/her decision (to call the physician, etc.) were assigned positive, negative or neutral points depending on the steps of the case. Moreover, positive or negative points were assigned to the quality of communication during the SBAR tool part of the game. The detailed score of case 1 is presented in Additional file 2.

### Study description

In this prospective, observational and non-interventional study, the participants were divided into three groups after giving informed consent.

- *Student nurse (S) group*: graduate nursing students at the end of their second year of training.
- *Recently graduated (R) group:* nurses having graduated less than 2 years before the study, who worked in a medical or surgical ward.
- *Expert nurse (E) group*: nurses having graduated more than 4 years before the study, who worked in an intensive care unit.

The gaming sessions were held at the LabForSIMS simulation centre at the Paris Sud Medical School and at the Sud Francilien Nursing School. Each volunteer played cases 1, 2 and 3 in randomised order on an individual computer.

### Validity evidence

The objective of this study was to assess the validity evidence of *LabForGames Warning* before using the game in educational activities.

At the beginning of our study and according to Graafland et al., the validity of a SG should be assessed by using content validity, face validity, construct validity, concurrent validity and predictive validity [15, 10, 18]. However, this classical validation framework may be replaced by those of Messick or Kane [14, 19]. To date, few studies in the simulation field have used the latter frameworks [19–22]. In their systematic review, Borgensen et al. reported that only 6.6% of the surgical simulation studies published up to 2017 used Messick’s recommended validity framework [21]. Moreover, only five studies have assessed all five domains of the Messick framework in the surgical studies reviewed. In the present study, the following five domains of Messick’s framework for validity evidence were assessed: content, response process, internal structure, relations to other variables and consequences [14].

Content is defined by “the relationship between the content of a test and the construct it is intended to measure” [14]. The educational content, learning objectives and branched steps were developed by clinical and pedagogical experts (nine instructors of four nursing schools and three anesthesiologists who were also simulation instructors) in conformity with the French nurse training curriculum [5] and literature review. For each scenario, the script, pedagogical objectives, feedback and scoring were written, reviewed and validated through expert consensus. Virtual clinical case development was also the product of iterative dialogues between the pedagogical team and the developer. Pilot testing involved pedagogical clinical experts (different from group E) and corrections were made before the final version was used in the study. Moreover, content validity in the study was assessed by expert nurses (group E) who judged the medical content and the educational objectives of the game (using a ten-point Likert scale).

The response process is “the fit between the construct and the detailed nature of performance [...] actually engaged in” [14]. During the SG sessions, we controlled the security (defined as the prevention of cheating) [14] and the quality of this assessment. All participants completed a standardised tutorial just prior to using the SG. Each participant played the game on an individual computer with no personal documents. An instructor was present at all times to prevent cheating. The instructors had no access to the scores. We also analysed the participants’ perception with the aid of a questionnaire at the end of the SG session. The following participant characteristics—sex, age, post-graduate experience, intensive care experience and previous video gaming activity (entertainment and professional education)—were recorded (Table 1). This questionnaire also assessed the participants’ perception of three main themes: satisfaction with the educational tool, game realism and future professional impact (using a ten-point Likert scale) (Table 2). Self-assessment of the clinical reasoning learning process was also recorded after the session. This questionnaire, translated into French, had previously been related by Koivisto et al. and assesses the various steps of clinical nursing reasoning as defined by Levett-Jones et al. [23, 24]. Each question assesses a specific step in the clinical reasoning process (“I learned to...”) with the use of a five-point Likert scale. The global result (graded out of 70) was obtained by totalling the values assigned to the 14 questions (Table 3).

**Table 1:** Characteristics of players included in group S (nursing students), group R (recently graduated nurses), and group E (expert nurses)

	Group S ***n*** = 25	Group R ***n*** = 25	Group E ***n*** = 21	***p***
**Age** (years)	25.0 ± 6.6	25.0 ± 4.2	31.6 ± 5.9^**,***^	0.0004*
**Sex F/M**	22 (88%)/3 (12%)	19 (76%)/6 (24%)	14 (67%)/7 (33%)	0.22
**Post-graduate professional experience** (years)	0	1.4 ± 0.5	7.9 ± 4.5^**,***^	0.0004*
**Previous ICU experience** (years)	0	0.04 ± 0.2	5.5 ± 3.9^**,***^	
**Video gaming activity**				0.40
^$^Never	17 (71%)	16 (64%)	14 (66.7%)	
1/month	5 (21%)	5 (20%)	5 (24%)	
1/week	2 (8%)	1 (4%)	2 (10%)	
Every day	0 (0%)	3 (12%)	0 (0%)	
**Video gaming activity in healthcare** (% of players)	1 (4%)	0 (0%)	4 (19%)^**,***^	0.03*
**Experience in specific situations** (1 [none] to 10 [expert])				
Post-operative haemorrhage	2.1 ± 1.7	2.8 ± 2.3	7.3 ± 1.5^**,***^	< .0001*
Brain trauma	2.1 ± 1.7	3.3 ± 2.6^****^	7.8 ± 1.4^**,***^	< .0001*
Intestinal obstruction	2.3 ± 1.8	2.6 ± 2.1	6.7 ± 1.9^**,***^	< .0001*

Results are reported as mean ± SD or as a percentage and compared using ANOVA test or chi^2^ test (followed by post hoc tests when significant)

*p value < 0.05 between groups S, R, and E

^**^Group E ≠ S with *p* < 0.05

^***^Group E ≠ group R with *p* < 0.05

^****^Group S ≠ group R with *p* < 0.05

^$^No response for one student

**Table 2:** Results of the self-questionnaire assessing response process validity evidence in the three groups

	Group S ***n*** = 25	Group R ***n*** = 25	Group E ***n*** = 21	***p***
**Q1. Do you think that this serious game provided a complete and good nursing care experience with respect to the medical content and educational objectives?** 1 (No, not at all) to 10 (completely correct and good)				
Post-operative haemorrhage scenario	7.8 ± 1.2	8.0 ± 1.3	7.8 ± 1.3	0.83
Brain trauma scenario	7.8 ± 1.3	7.0 ± 1.3	8.2 ± 1.0	0.48
Obstructed intestinal tract scenario	7.5 ± 1.4	7.7 ± 1.5	7.5 ± 1.4	0.86
**Q2. Do you think that this serious game was realistic?** 1 (not realistic) to 10 (very realistic)	7.7 ± 1.4	7.0 ± 1.6	7.5 ± 1.4	0.25
**Q3. Do you think that the clinical scenario was realistic?** 1 (not realistic) to 10 (very realistic)				
Post-operative haemorrhage scenario	8.0 ± 2.0	7.7 ± 1.6	8.2 ± 1.1	0.50
Brain trauma scenario	8.1 ± 1.4	7.8 ± 1.3	8.6 ± 0.7	0.07
Obstructed intestinal tract scenario	7.4 ± 2.0	7.6 ± 1.4	8.0 ± 1.1	0.39
**Q4. Do you think that the history-taking was realistic?** 1 (not realistic) to 10 (very realistic)	7.9 ± 1.3	7.4 ± 1.4	7.5 ± 1.2	0.41
**Q5. Do you think that the clinical exam was realistic?** 1 (not realistic) to 10 (very realistic)				
Post-operative haemorrhage scenario	7.8 ± 1.2	7.7 ± 1.4	8.1 ± 1.0	0.53
Brain trauma scenario	8.0 ± 1.0	7.8 ± 1.3	8.1 ± 1	0.58 0.76
Obstructed intestinal tract scenario	7.5 ± 1.5	7.8 ± 1.3	7.8 ± 1.1	
**Q6. Do you think that the care report was realistic?** 1 (not realistic) to 10 (very realistic)	8.5 ± 1.0^**,****^	7.5 ± 1.6	7.4 ± 1.9	0.02*
**Q7. Do you think that the graphics and animations were realistic?** 1 (not realistic) to 10 (very realistic)	8.4 ± 0.9	7.6 ± 1.3	7.8 ± 1.7	0.06
**Q8. Overall, are you satisfied with this serious game as an educational tool?** 1 (not satisfied) to 10 (very satisfied)	7.0 ± 1.5	7.0 ± 1.7	7.0 ± 1.5	0.98
**Q9. Do you think that incorporating the serious game concept improves your training?** 1 (No, not at all) to 10 (Yes, very much)	8.4 ± 1.2	7.7 ± 2	6.9 ± 2.9	0.05
**Q10. Do you think that this tool increases your skills?** 1 (No, not at all) to 10 (Yes, very much)	8.4 ± 1.5	7.7 ± 1.8	5.5 ± 2.1^**,***^	< .0001*
**Q11. Do you think that this training will have an impact on your professional work?** 1 (No, not at all) to 10 (Yes, very much)	7.3 ± 1.3	6.8 ± 1.8	4.4 ± 1.9^**,***^	< .0001*
**Q12. Would you recommend this training to other students or colleagues?** 1 (No, not at all) to 10 (Yes, very much)	8.8 ± 1.2	8.2 ± 1.5	8.1 ± 1.8	0.17

Results are described as means ± SD and compared using ANOVA (followed by post hoc comparison when significant)

^**^Group E ≠ S with *p* < 0.05

^***^Group E ≠ group R with *p* < 0.05

^****^Group S ≠ group R with *p* < 0.05

**Table 3:** Results of self-assessment of learning the clinical reasoning process between groups

I learned: 1 (not at all) to 5 (very much)		Group S ***n*** = 24	Group R ***n*** = 25	Group E ***n*** = 21	***p***
**To collect information**	Collect information by interviewing patient	4 ± 0.6	4.2 ± 0.7	4 ± 0.8	0.57
	Collect information by observing patient	3.7 ± 0.7	4.1 ± 0.7	3.9 ± 0.9	0.24
	Collect information from measurable patient data	3.9 ± 0.8	4.2 ± 0.7	4 ± 0.8	0.45
**To process information**	Analyse data to reach an understanding of signs or symptoms	4.1 ± 0.7	4.0 ± 0.7	3.9 ± 0.7	0.69
	Distinguish between relevant and irrelevant information	3.7 ± 0.8	3.7 ± 0.7	3.8 ± 0.8	0.96
**To identify problems/issues**	Make nursing diagnoses	3.8 ± 0.8	4 ± 0.8	3.7 ± 0.8	0.61
	Take decisions on patient care independently	3.4 ± 0.9	3.9 ± 0.6	3.7 ± 0.9	0.17
	Take decisions on patient care in cooperation with other students	2.6 ± 1.0	3.3 ± 1.0	3.5 ± 0.9	0.01^£^
	Take decisions on patient care promptly	3.5 ± 0.8	3.72 ± 0.8	3.9 ± 0.9	0.27
**To establish goals**	Prioritise patient’s needs for care	3.4 ± 1.1	3.9 ± 0.7	3.7 ± 1.0	0.21
	Set goals	3.3 ± 0.8	3.9 ± 0.6	3.5 ± 1.0	0.02^£^
	Plan nursing interventions	3.7 ± 0.9	3.9 ± 0.7	3.5 ± 0.9	0.23
**To take action**	Implement nursing interventions	3.8 ± 1.0	4.0 ± 0.8	3.5 ± 1.1	0.35
**To evaluate outcomes**	Evaluate the effectiveness of interventions	3.5 ± 1.0	3.6 ± 0.8	3.4 ± 1.1	0.69
**Total score (/70)**		50.8 ± 6.4	54.8 ± 6.4	52.2 ± 9.5	0.17

Results are described by means ± SD and compared using ANOVA test (followed by post hoc comparison when significant). One student was excluded for not answering the clinical reasoning self-questionnaire. The questionnaire, translated in French, describes the steps of clinical nursing reasoning as defined previously ^23 24^

^£^Bonferroni criterion: alpha/14 = 0.0035 to be significative

The internal structure is defined by “the relationship among data items within the assessment and how these relate to the overarching construct” [14]. A factor analysis (principal component analysis) was used to identify the relations between the main steps of the clinical reasoning process (using the data from the self-assessment questionnaire presented in Table 3) [23, 24] and the non-technical errors (situational awareness, communication) at each level of expertise (S, R and E groups). Concerning errors, negative points were classified as situational awareness errors when they related to the diagnostic part of the scenario and as communication errors when they occurred during the SBAR tool part of the game.

Relations to other variables are the “degree to which these relationships are consistent with the construct underlying the proposed test score interpretations” [14]. The ability of this SG to measure differences between groups of different skill levels was assessed by comparing the scores and the playing time of groups S, R and E. The scores obtained for each case were graded out of 100 points. The playing time in each case (in minutes) was also assessed.

Consequences are “the impact, beneficial or harmful and intended or unintended, of assessment” [14]. A posteriori, we identified the results of examinations related to training sequences that were associated with the SG pedagogical objectives: “care project module,” “emergency module,” and “plan and implement nursing interventions and therapeutics module.” We then compared the exam results obtained by group S participants and those of students who had not participated in the study (i.e. the remaining students in the same class who did not participate in the SG session).

### Statistical analysis

Game scores were used to define the number of participants to be included. Considering that group S would obtain a novice score (no reference available but estimated at 60/100) and that group E would have an approximate score of 80/100 (no reference available), the difference between the students and the experts was 20/100. Considering a standard deviation of 15 points, the sample size was 12 per group with the use of a two-tailed analysis (alpha risk = 0.05 and power of 0.9) [25]. In view of the risk of attrition, we decided to form groups of 20 participants.

The results are presented as means ± standard deviation or percentages and confidence intervals. After the normal distribution assessment, statistical analysis was performed using parametric tests (one-way ANOVA test or chi [2] test, followed by post hoc tests in the case of significant comparison) (JMP software, SAS Institute ®). The factorial analysis (principal component analysis) was performed using Statistica software (StatSoft Inc. ®). A *p* value less than 0.05 was considered significant, and adjustment for multiple comparisons was performed.

### Ethical statement

This study was approved (on March 30, 2017) by the Institutional Review Board of Paris Saclay University (CERNI). The project has been registered on ClinicalTrials.gov (ClinicalTrials.gov ID: NCT03092440) [26]. The study was conducted with the use of the CONSORT tool adapted for simulation studies and the GREET Tool for educational studies [27].

## Results

### Inclusion

Seventy-one nurses and nursing students participated in this study voluntarily between March and September 2017. Participants in group S were students at the Sud Francilien nursing school, whereas graduated nurses were recruited at the Kremlin Bicêtre University teaching hospital (group R from medical and surgical units and group E from two ICUs). Participant characteristics are presented in Table 1. One student experienced a technical problem during case 1 so no data could be stored for the analysis of case 1. Another student failed to record the clinical reasoning self-assessment. All of the participants played all three cases to the end.

### Content evidence

The nurses in group E considered the SG as providing complete and good nursing care regarding the medical content and educational objectives for the three cases (Q1) (Table 2). The global educational value of this SG was also positively perceived by group E (Q8-9).

### Response process evidence

A summary of the perception survey is shown in Table 2. All three groups scored the realism and graphics of the three scenarios positively with no significant difference between the groups (Q2-Q5, Q7). Group E considered the care record (Q6) less realistic than did the other two groups (*p* < 0.05).

The global educational value of the SG was perceived positively with no significant difference by all three groups (Q8-9). Groups S and R declared that the game could improve their skills (Q10) and could have an impact on their professional work (Q11). Conversely, group E perceived the game as less useful in improving their practice (*p* < 0.05) (Q10-11). However, all three groups stated they would recommend this session to students or colleagues (Q12).

Following training with the SG, all participants considered that their knowledge of the different steps of the clinical reasoning process had increased (self-assessment). There was no significant difference in the group scores (Table 3).

### Internal structure evidence: factor analysis

**Fig. 2:**
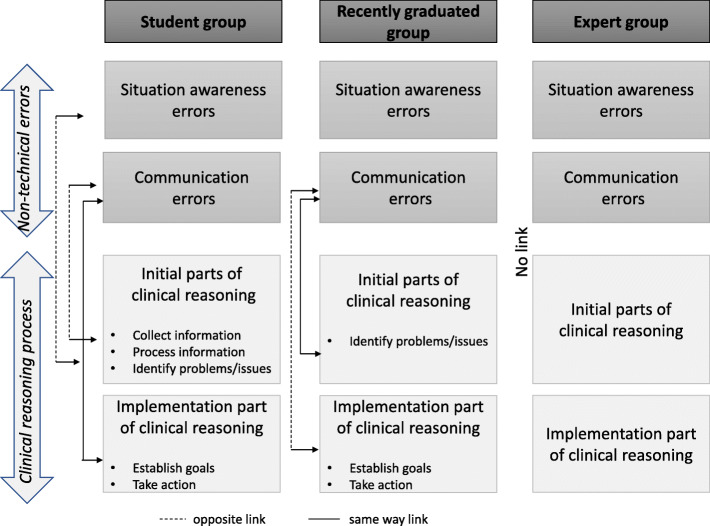
Links between errors (communication and situational awareness) and the clinical reasoning process as demonstrated by principal component factor analysis

Factor analysis was used to confirm the validity of the self-reporting questionnaire and to distinguish between the factors studied (realism, educational content and impact on the participant) (Additional file 3: Table S2). Factor analysis was also used to identify relations between the clinical reasoning process and errors (communication and situational awareness) in the groups (Fig. 2 and Additional file 3: Table S3). In group S, the first part of clinical reasoning (collect/process/identify) was linked to both situational awareness and communication errors whereas the implementation part (establish goal/take action) was linked to communication errors only. In group R, only communication was found to be related to the first part of reasoning (identify) on the one hand, and the implementation part (decision/treatment) on the other. In group E, no relation could be observed between clinical reasoning and errors of communication or situational awareness.

### Evidence regarding relations to other variables: comparison of scores and playing time between groups

There was no significant difference in scores between groups (main outcome), and no significant difference was found in the playing time between groups (Table 4). Moreover, no correlation between individual scores and playing time was observed between groups or for the whole set of participants (case 1: *r* = − 0.08, *p* = 0.48; case 2: *r* = 0.06, *p* = 0.61; case 3: *r* = − 0.10, *p* = 0.43); nor did factor analysis demonstrate any relationship between the scores and participants’ experience (Additional file 3: Table S1 (a)). Moreover, no relationship was observed between the scores and questions about content and face validity (Additional file 3: Table S1 (b)).

**Table 4:** Scores and playing time for the three groups

	Group S ***n*** = 25	Group R ***n*** = 25	Group E ***n*** = 21	***p***
**Scores** (score: 50 to 100)				
Post-operative haemorrhage scenario	45.5 ± 24.7 ^£^	48.0 ± 19.6	41.9 ± 28.9	0.69
Brain trauma scenario	31.9 ± 31.3	38.4 ± 38	39.1 ± 44.5	0.77
Obstructed intestinal tract scenario	42.6 ± 21.6	33.7 ± 22.9	45.7 ± 21.5	0.16
**Playtime** (minutes)				
Post-operative haemorrhage scenario	25.7 ± 6.3 ^£^	24.9 ± 7.3	25.6 ± 9.6	0.92
Brain trauma scenario	33.5 ± 7.7	28.5 ± 6.5	31.7 ± 10.5	0.10
Obstructed intestinal tract scenario	30 ± ± 7.3	29.4 ± 9.0	28.3 ± 7.4	0.75

Results are described by means ± SD and compared using ANOVA (followed by post hoc comparison when significant). The participant’s actions were assigned neutral, negative or positive points, as defined by the pedagogical team. The score and playing time were generated automatically by the serious game software

*p value < 0.05 was considered significant between groups S, R, and E

^£^*n* = 24 because one student experienced a technical problem with data recording in case 1 which could not be stored for analysis

### Evidence for consequences

A posteriori analysis demonstrated no significant difference in the three exam results (graded/20) between group S (*n* = 25) and the control group (*n* = 111) (care project module: 14.3 ± 2.4 vs 13.9 ± 2.3, *p* = 0.41; emergency module: 10.8 ± 1.8 vs 10.6 ± 2.3, *p* = 0.68; and plan and implement nursing interventions and therapeutics module: 13.8 ± 3.4 vs 12.4 ± 3.1, *p* = 0.07, respectively).

## Discussion

Validity assessment is necessary for an SG, as for any new educational tool [14, 15]. In this study, we used the five domains of validity evidence described by Messick et al. [14] (content, response process, internal structure, relations to other variables and consequences). The main findings are that neither the gameplay scores nor the playing time of *LabForgames Warning* differentiated the level of the nurses’ skills. However, other domains of validity evidence for this SG were demonstrated.

First, content validity evidence is the most frequently assessed domain in educational literature [14, 28–31]. *LabForGames Warning* (educational content and objectives, different branched steps, scoring) was produced by clinical, pedagogical and interprofessional experts in conformity with the French nurse training curriculum [5] and literature review. Effective educational content was demonstrated as experts (group E) expressed a positive attitude toward the medical algorithm and the nurse decision-making process, confirming content legitimacy.

A second domain of validity evidence was the response process, which was assessed using rigorous quality and security control during the study. Moreover, both experts and novices were asked to assess the tool’s apparent similarity with reality and its usefulness for educational purposes. Evaluation by experts is especially crucial in order to collect validity evidence. In our study, the experts were from the units in which the game’s cases took place (orthopaedic department and psychiatry department but not from the nursing home). Moreover, nurses work in many different units (surgery, medicine, etc.) prior to graduating. Realism was considered for the whole gameplay but also for its different parts (i.e. nursing care, clinical examination, care records and graphics). The difference found for care record realism between groups may be explained by the fact that electronic care records are not available in all hospital units, which complicates extrapolation. Moreover, the SG’s ability to improve skills, or the impact on the professional outcome, were evaluated positively, especially by students and recently graduated nurses, confirming our initial educational choice to target this population.

Satisfaction with the training process, skill improvement self-assessment and the impact on professional outcomes were considered satisfactory. Moreover, after training with the SG, all of the participants felt that their skills had improved in the different steps of the nurse clinical reasoning process, with a global score of 52/70. Teaching clinical reasoning with the aid of an SG appears to be of value and relevant for trainees. The virtual cases represent experiential learning as described by Kolb [32] and explore the four domains of the clinical reasoning process [33]. Learning of clinical reasoning is complex to assess [34], and although self-assessment involves only a subjective perception, it does provide important information. The tool we used was based on the clinical reasoning process described by Levett-Jones and used by Koivisto [24, 23]. Other tools have also been published [35, 36]. Despite their uncertain validity, these tools aim to assess the various steps of clinical reasoning. However, most studies have analysed only the results of the overall reasoning process (i.e. diagnosis and treatment) but not all of the steps of clinical reasoning [8, 37–40].

Third, with regard to validity evidence for internal structure, factor analysis appeared to be a useful tool to identify behaviours specific to each group by assessing the relations between parts of the clinical reasoning process and errors. Clinical reasoning is a complex cognitive process [41]. According to the dual-process theory, two cognitive systems are used by healthcare providers. System 1 is heuristic reasoning based on illness pattern recognition (matching an actual configuration of signs with previously encountered equivalent situations), allowing intuitive mental shortcuts to reduce the cognitive load of decision making. System 2 is an analytical reasoning model that integrates all available information and requires great effort. Simply stated, system 1 is more easily implemented by experts due to clinical experience whereas system 2 would be used more often by novices. Interestingly, in our study, organisation of links between parts of the clinical reasoning process and errors was found to be an indicator of expertise. In group E, no relation between clinical reasoning, communication and situation awareness was observed, suggesting that encapsulation of clinical reasoning occurs with experience and is congruent with a more frequent use of system 1 (intuitive) processing [41]. Each step is dependent on the following one, as a unique “module” of clinical reasoning [42]. Moreover, the independence of the clinical reasoning items of the self-assessment suggests that the modularity of clinical reasoning is embedded in a deeper structure that is inaccessible to awareness and with no explicit link. On the contrary, in group S, the first part of reasoning was linked to both situational awareness and communication errors whereas the implementation part was linked to communication errors only. In group R, only communication was found to be related positively to the first part of reasoning and negatively to the implementation part, which suggests a beginning of expertise. However, although we did not assess situation awareness itself using validated methods [43], we classified errors (negative points) occurring in the diagnostic part of the scenario and those related to decisions regarding the next monitoring interval as “situation awareness” errors and investigated relations with other variables through factor analysis. Moreover, we did not record the exact time at which each action was done during each step. Furthermore, we do not know precisely when deterioration was identified since situational awareness is a progressive process with interconnecting steps*.* Only the global playtime of cases was recorded with no significant difference between groups. Therefore, individualization of the different steps of the clinical reasoning process during the game was not possible and the manner in which participants performed individually in each case could not be determined. It could be interesting to introduce markers for each step of the clinical reasoning process in a future version of the game.

The fourth domain of validity evidence studied was the relation to other variables by comparing the groups’ scores and playing time. In previous studies, the validity of the scoring system was either not assessed [24, 44–46] or was assessed only by means of “static” multiple choice questions that were obvious to the participant since they represented the logical steps of a surgical procedure [28–30]. In contrast, our game design was branched and included algorithms with many possibilities and different interactions between the patient and the physician, as the various ramifications in the storyboard aimed to reproduce the most likely clinical situations. Our SG combined the assessment of several non-technical skills, including situational awareness and communication. Therefore, our results highlight the difficulty in establishing a scoring system due to several interactive and complex problems. In a similar SG in critical paediatric emergency care, Gerard et al. demonstrated validity evidence for *Pediatric Sim* Game scores with higher scores for attendings followed by residents than for medical students [20]. A strong positive correlation was found between game scores and written knowledge test scores. However, as with our SG (Table 4), game scores were low across all groups (68/100 for attendings), which confirm the difficulty in constructing a score.

Additionally, the score explores only a limited part of the tool and not all of its pedagogical impact and utility. The assessment of construct validity is essential if the SG is to be used in a summative educational process. Indeed, if the score’s construct validity is not demonstrated, the game cannot be used to evaluate student learning at the end of an instructional unit. To our knowledge, no SG like this one has been used for summative assessment. However, this version of our SG can be used for training in an educational programme since some domains of validity evidence could be demonstrated. Certain teams have already included an SG in their training programme [24, 44, 47]. The nursing school participating in this project recently introduced it in the student curriculum because the detailed scoring analysis can improve the instructors’ debriefing since they can use the detailed scoring of each trainee, available on an e-platform. More studies are necessary to define the place of this tool in professional healthcare education.

Since *LabForGames Warning* scores could not differentiate between the levels of expertise, one might wonder how it might be improved. The instructors tried to align the scoring system to case complexity, for which a certain level of proficiency was expected. In the post-operative haemorrhage case, for example, analysis of the detailed subscores showed that the majority (> 85%) of essential nursing care actions were performed by each group. Participant actions were stereotyped and limited. Allocating points in a different manner and/or increasing the number of tasks available to the participant including some unnecessary (or even deceptive) actions might be more discriminant. Indeed, one limitation of the game itself is that if the participant performs all actions, many positive points may be earned with no actual clinical reasoning. Recording the response time at each point could also be useful.

Playing time is a surrogate marker of the time it takes to care for the patient, collect data, make a decision and call for help. Although one might expect playing time to be longer for the novice than for the expert, this study found no difference in the playing time between the groups. Results of previous studies are mixed on this subject and do not consistently show a direct correlation between playing time and expertise [28, 48]. The absence of differences in playing time could be explained by the fact that the time devoted to each participant’s action and communication is limited and predefined by the game itself.

When trying to study the consequences of using the SG (i.e. the last domain of validity evidence), a posteriori analysis found no significant difference in examination results between the student group having played the SG and the control group. However, no definitive conclusion can be drawn since the groups studied were not randomised.

One limitation of the study was that several items of our set of measures to assess validity were based on participant perception, although objective measures would appear more potent. Perception, however, is more often studied in the literature. For example, Graafland et al. and Sugand et al. validated the content and face validity of their SG with a self-report questionnaire [28–31].

Another limitation was that mean scores were low (< 50/100). Some negative points attached to the communication part were based on the use of the SBAR tool as even our expert nurses had received no previous training on how to use this tool for which a French version had only recently been made available [5]. However, even when scores were recalculated after excluding the SBAR tool, no significant differences were observed between groups.

Writing gameplay is a difficult task since each clinical situation has several possible outcome branches and poor writing may lead to low score results and poor discrimination. However, the pedagogical team was composed of medical experts (anaesthesiologists) and nursing experts, who were also experts in pedagogy. Interestingly, all of the teachers were also experienced high-fidelity session instructors.

## Conclusions

In conclusion, our study demonstrated that the scores and the playing time of the game *LabForGames Warning* did not differentiate nurses’ levels of clinical skills. However, validity evidence was obtained from the content, the response process and the internal structure. Although the present version cannot be used for the summative assessment of nursing students, our study has shown that this SG was well received by the participants and that it can be used for training in an educational programme. More studies are necessary to improve SG scoring details, and, more generally speaking, to define this new tool’s place in the field of education.

## Supplementary information

**Additional file 1: ** Screenshot of *LabForGames Warning* serious game.

**Additional file 2:Case 1.** Post-operative haemorrhage and score grids. (PPTX 64 kb)

**Additional file 3:Table S1.** Factorial analysis (Principal Component Analysis, PCA) (a) for scores and players’ experience and (b) for scores and questions about content validity and face validity (see scores and questions in Tables 2 and 3). **Table S2.** Factorial analysis (Principal Component Analysis, PCA) testing various aspects of the questionnaire (realism, educational content and impact on the player) (scores and questions in Tables 2 and 3). **Table S3.** Factorial analysis (Principal Component Analysis, PCA) between the clinical reasoning process components (self-questionnaire) and errors made during the game (communication and situational awareness). Two students were excluded (one because data on the post-operative haemorrhage scenario were lacking and another because he/she did not answer the questionnaire).

## Data Availability

The datasets used and/or analysed during the current study are available from the corresponding author on reasonable request.

## Abbreviations

S: Student nurse
R: Recently graduated
E: Expert nurse
SG: Serious game
