# Supplementary material for: Assessing validity evidence for a serious game dedicated to patient clinical deterioration and communication
Source: Adv Simul (Lond). 2020 May 27;5:4. doi: 10.1186/s41077-020-00123-3 (PMC7251894; doi:10.1186/s41077-020-00123-3)
Supplement: Supplementary file 2 — Additional file 2:Case 1. Post-operative haemorrhage and score grids. (PPTX 64 kb) [file 41077_2020_123_MOESM2_ESM.pptx]

## Slide 1
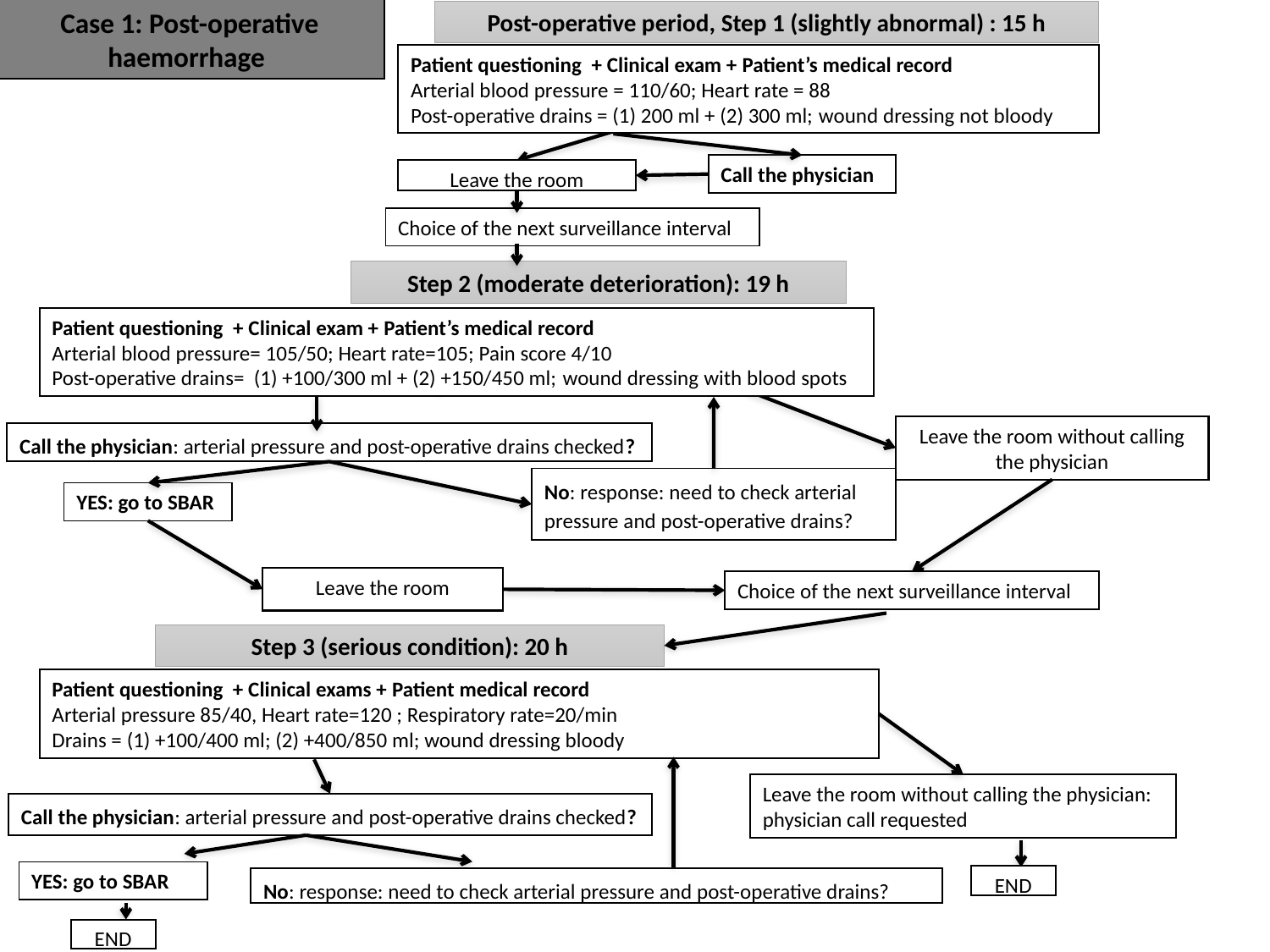

Case 1: Post-operative haemorrhage
Post-operative period, Step 1 (slightly abnormal) : 15 h
Patient questioning + Clinical exam + Patient’s medical record
Arterial blood pressure = 110/60; Heart rate = 88
Post-operative drains = (1) 200 ml + (2) 300 ml; wound dressing not bloody
Call the physician
Leave the room
Choice of the next surveillance interval
Step 2 (moderate deterioration): 19 h
Patient questioning + Clinical exam + Patient’s medical record
Arterial blood pressure= 105/50; Heart rate=105; Pain score 4/10
Post-operative drains= (1) +100/300 ml + (2) +150/450 ml; wound dressing with blood spots
Leave the room without calling the physician
Call the physician: arterial pressure and post-operative drains checked?
No: response: need to check arterial pressure and post-operative drains?
YES: go to SBAR
Leave the room
Choice of the next surveillance interval
Step 3 (serious condition): 20 h
Patient questioning + Clinical exams + Patient medical record
Arterial pressure 85/40, Heart rate=120 ; Respiratory rate=20/min
Drains = (1) +100/400 ml; (2) +400/850 ml; wound dressing bloody
Leave the room without calling the physician:
physician call requested
Call the physician: arterial pressure and post-operative drains checked?
YES: go to SBAR
END
No: response: need to check arterial pressure and post-operative drains?
END

## Slide 2
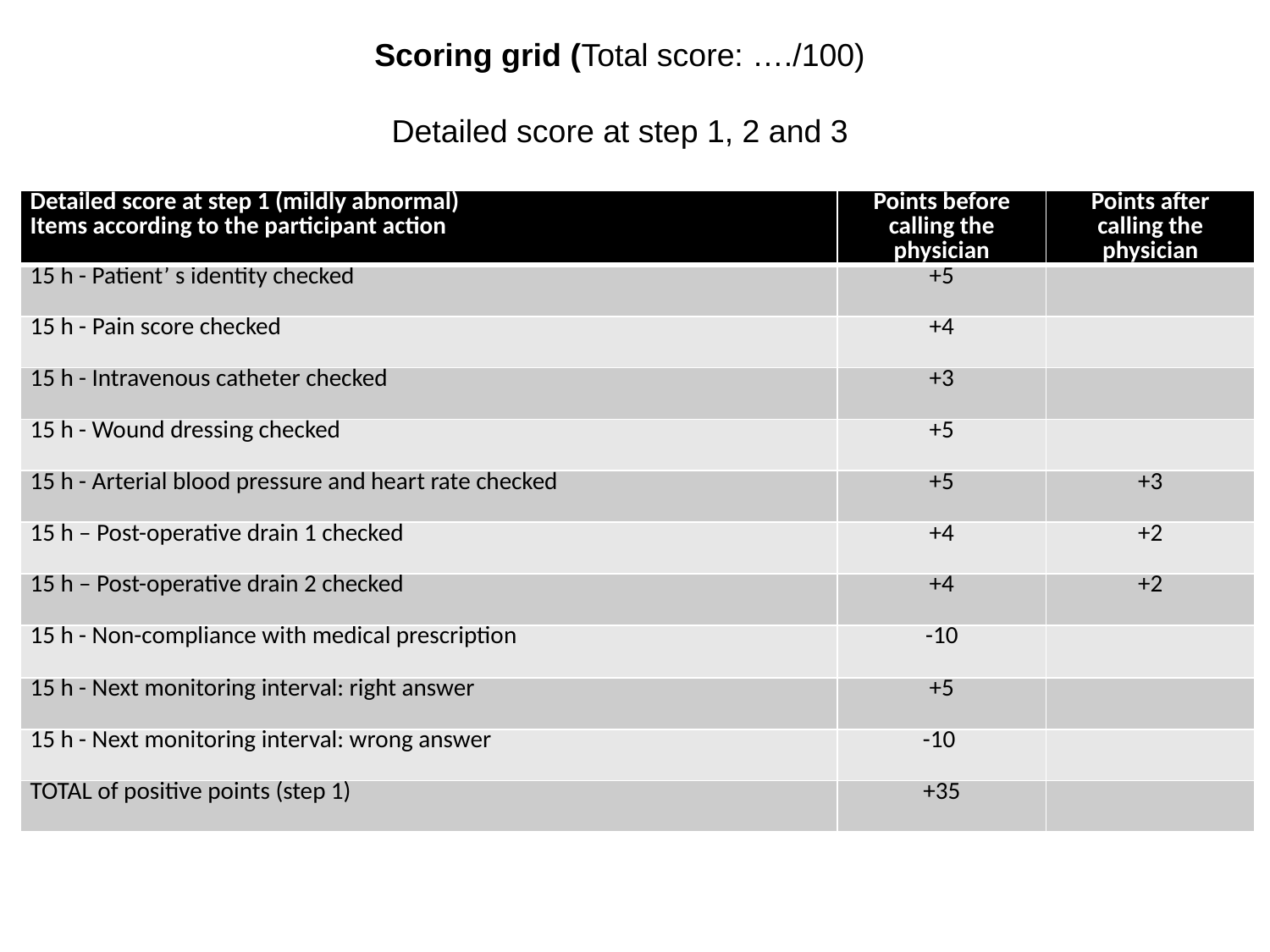

Scoring grid (Total score: …./100)
Detailed score at step 1, 2 and 3
| Detailed score at step 1 (mildly abnormal) Items according to the participant action | Points before calling the physician | Points after calling the physician |
| --- | --- | --- |
| 15 h - Patient’ s identity checked | +5 | |
| 15 h - Pain score checked | +4 | |
| 15 h - Intravenous catheter checked | +3 | |
| 15 h - Wound dressing checked | +5 | |
| 15 h - Arterial blood pressure and heart rate checked | +5 | +3 |
| 15 h – Post-operative drain 1 checked | +4 | +2 |
| 15 h – Post-operative drain 2 checked | +4 | +2 |
| 15 h - Non-compliance with medical prescription | -10 | |
| 15 h - Next monitoring interval: right answer | +5 | |
| 15 h - Next monitoring interval: wrong answer | -10 | |
| TOTAL of positive points (step 1) | +35 | |

## Slide 3
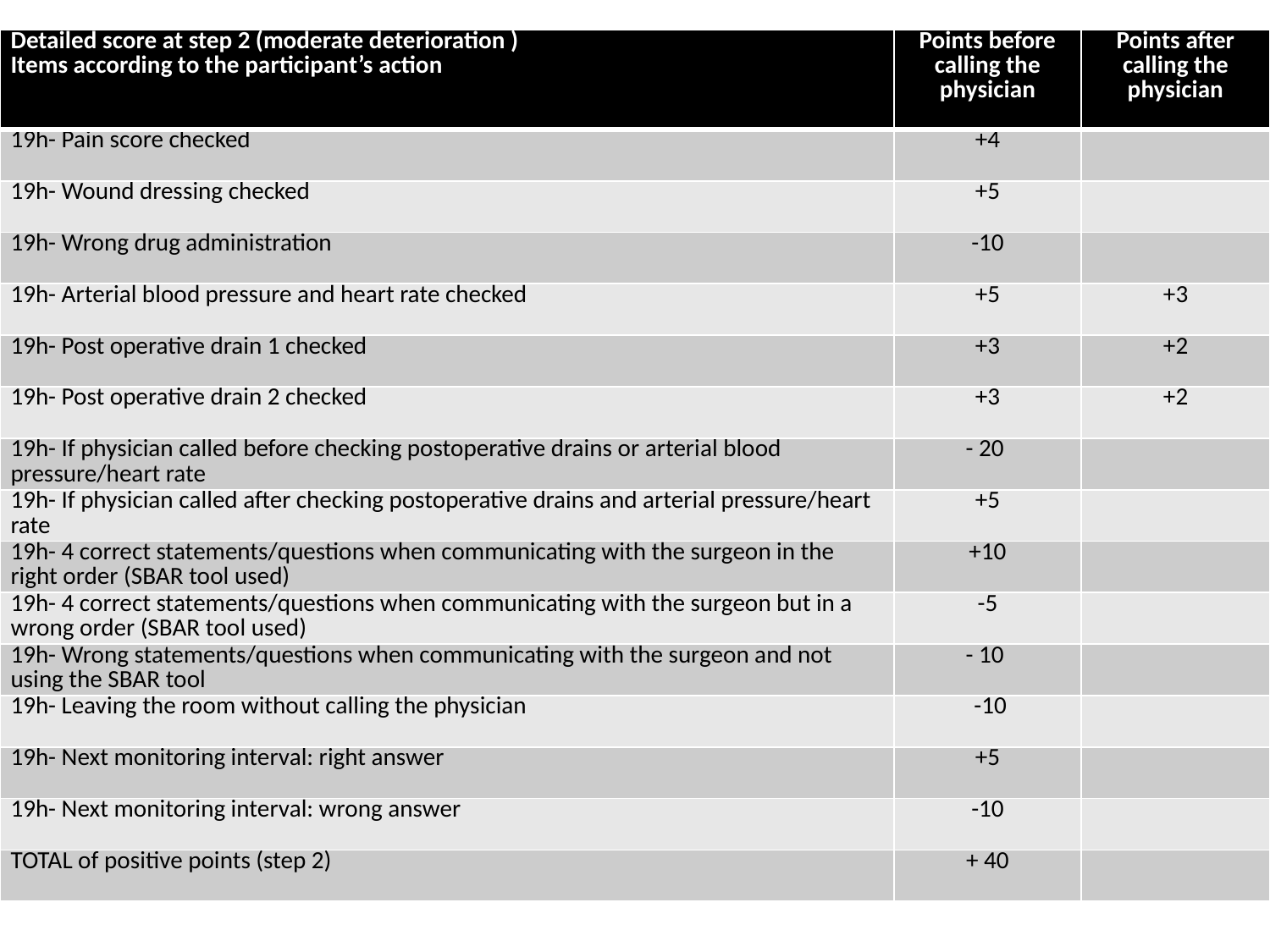

| Detailed score at step 2 (moderate deterioration ) Items according to the participant’s action | Points before calling the physician | Points after calling the physician |
| --- | --- | --- |
| 19h- Pain score checked | +4 | |
| 19h- Wound dressing checked | +5 | |
| 19h- Wrong drug administration | -10 | |
| 19h- Arterial blood pressure and heart rate checked | +5 | +3 |
| 19h- Post operative drain 1 checked | +3 | +2 |
| 19h- Post operative drain 2 checked | +3 | +2 |
| 19h- If physician called before checking postoperative drains or arterial blood pressure/heart rate | - 20 | |
| 19h- If physician called after checking postoperative drains and arterial pressure/heart rate | +5 | |
| 19h- 4 correct statements/questions when communicating with the surgeon in the right order (SBAR tool used) | +10 | |
| 19h- 4 correct statements/questions when communicating with the surgeon but in a wrong order (SBAR tool used) | -5 | |
| 19h- Wrong statements/questions when communicating with the surgeon and not using the SBAR tool | - 10 | |
| 19h- Leaving the room without calling the physician | -10 | |
| 19h- Next monitoring interval: right answer | +5 | |
| 19h- Next monitoring interval: wrong answer | -10 | |
| TOTAL of positive points (step 2) | + 40 | |

## Slide 4
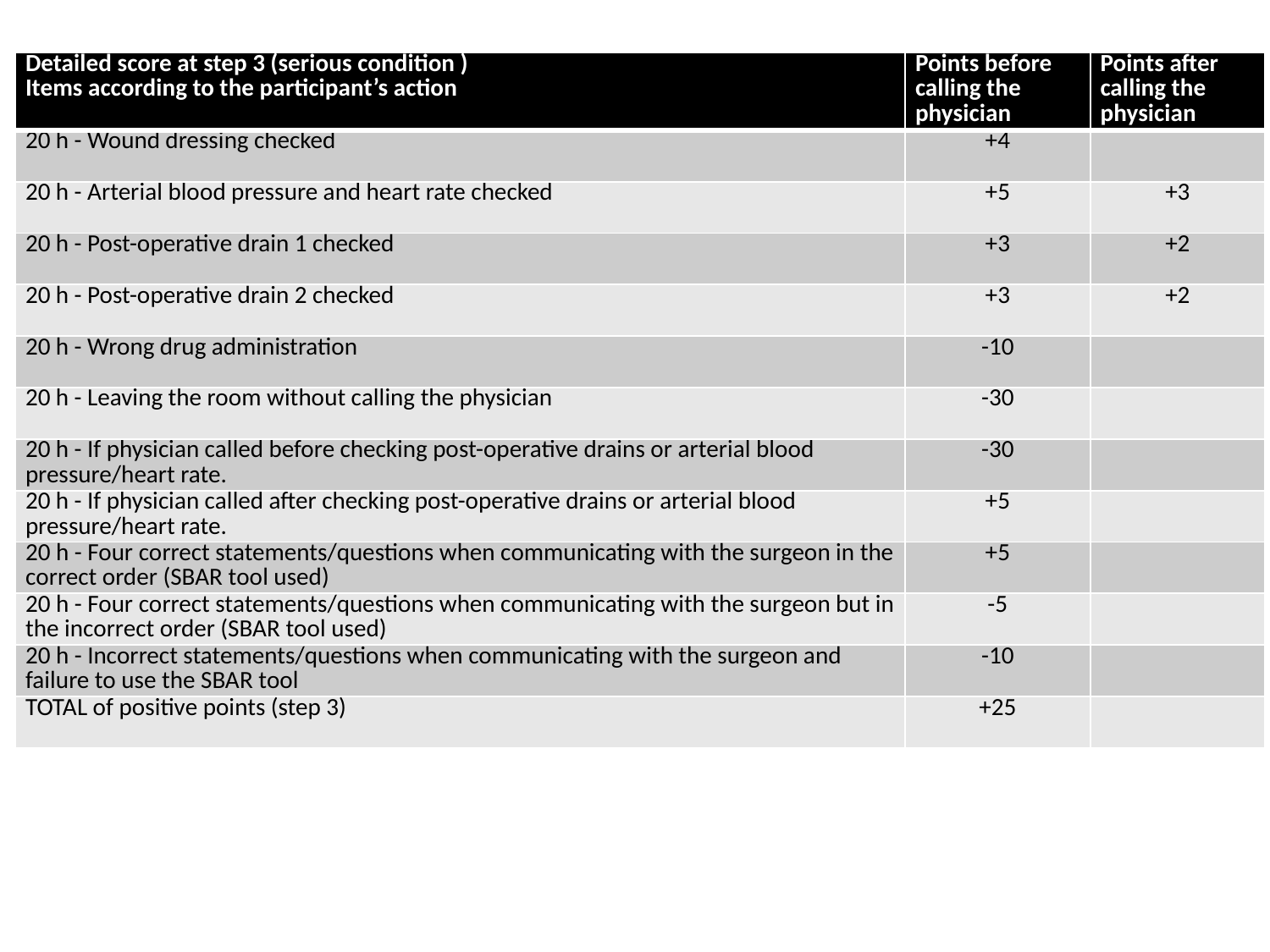

| Detailed score at step 3 (serious condition ) Items according to the participant’s action | Points before calling the physician | Points after calling the physician |
| --- | --- | --- |
| 20 h - Wound dressing checked | +4 | |
| 20 h - Arterial blood pressure and heart rate checked | +5 | +3 |
| 20 h - Post-operative drain 1 checked | +3 | +2 |
| 20 h - Post-operative drain 2 checked | +3 | +2 |
| 20 h - Wrong drug administration | -10 | |
| 20 h - Leaving the room without calling the physician | -30 | |
| 20 h - If physician called before checking post-operative drains or arterial blood pressure/heart rate. | -30 | |
| 20 h - If physician called after checking post-operative drains or arterial blood pressure/heart rate. | +5 | |
| 20 h - Four correct statements/questions when communicating with the surgeon in the correct order (SBAR tool used) | +5 | |
| 20 h - Four correct statements/questions when communicating with the surgeon but in the incorrect order (SBAR tool used) | -5 | |
| 20 h - Incorrect statements/questions when communicating with the surgeon and failure to use the SBAR tool | -10 | |
| TOTAL of positive points (step 3) | +25 | |
