# Supplementary material for: Assessing validity evidence for a serious game dedicated to patient clinical deterioration and communication
Source: Adv Simul (Lond). 2020 May 27;5:4. doi: 10.1186/s41077-020-00123-3 (PMC7251894; doi:10.1186/s41077-020-00123-3)
Supplement: Supplementary file 3 — Additional file 3:Table S1. Factorial analysis (Principal Component Analysis, PCA) (a) for scores and players’ experience and (b) for scores and questions about content validity and face validity (see scores and questions in Tables 2 and 3). Table S2. Factorial analysis (Principal Component Analysis, PCA) testing various aspects of the questionnaire (realism, educational content and impact on the player) (scores and questions in Tables 2 and 3). Table S3. Factorial analysis (Principal Component Analysis, PCA) between the clinical reasoning process components (self-questionnaire) and errors made during the game (communication and situational awareness). Two students were excluded (one because data on the post-operative haemorrhage scenario were lacking and another because he/she did not answer the questionnaire). [file 41077_2020_123_MOESM3_ESM.docx]

**Appendix 3**

**a)** Factorial analysis (Principal Component Analysis, PCA).

(a) for scores and players’ experience

(b) for scores and questions about content validity and face validity (see scores and questions in Tables 2 and 3).

* indicates loadings > 0.7 (i.e. the factor explains >49% of the item variance)

Commonalities estimate the proportion of variance of an item explained by all factors.

(a)

|  | **Factor 1** | **Factor 2** | **Commonalities**  **h²** |
| --- | --- | --- | --- |
| **Experience with post-operative haemorrhage** | **0.958*** | 0.062 | 0.922 |
| **Experience with brain trauma** | **0.938*** | 0.038 | 0.881 |
| **Experience with intestinal obstruction** | **0.907*** | -0.053 | 0.825 |
| **Score for post-operative haemorrhage scenario** | -0.105 | **0.742*** | 0.562 |
| **Score for brain trauma scenario** | 0.043 | **0.821*** | 0.676 |
| **Score for obstructed intestinal tract scenario** | 0.097 | **0.728*** | 0.539 |
|  |  |  |  |
| **Explained variance** | 44.1% | 29.4% | 73.5% |

(b)

| Scores and questions of Tables 2 and 3 | **Factor 1** | **Factor 2** | **Factor 3** | **Factor 4** | **Factor 5** | **Communalities**  **h²** |
| --- | --- | --- | --- | --- | --- | --- |
| **Q1 (post-operative haemorrhage scenario)** | **0.711*** | 0.078 | 0.190 | 0.247 | -0.101 | 0.619 |
| **Q1 (brain trauma scenario)** | **0.806*** | 0.222 | 0.056 | 0.059 | 0.224 | 0.756 |
| **Q1 (obstructed intestinal tract scenario)** | **0.821*** | 0.139 | 0.046 | -0.016 | -0.109 | 0.708 |
| **Q (easy to use the game)** | 0.262 | 0.233 | 0.117 | **0.713*** | 0.226 | 0.696 |
| **Q2** | 0.155 | 0.468 | 0.445 | -0.186 | 0.234 | 0.530 |
| **Q3 (post-operative haemorrhage scenario)** | 0.144 | 0.130 | **0.804*** | 0.151 | 0.116 | 0.720 |
| **Q3 (brain trauma scenario)** | 0.166 | 0.023 | **0.810*** | 0.157 | 0.110 | 0.721 |
| **Q3 (obstructed intestinal tract scenario)** | 0.194 | 0.011 | **0.792*** | 0.058 | 0.086 | 0.676 |
| **Q4** | 0.632 | 0.186 | 0.122 | 0.178 | -0.156 | 0.505 |
| **Q5 (post-operative haemorrhage scenario)** | 0.579 | -0.004 | 0.593 | 0.208 | 0.008 | 0.730 |
| **Q5 (brain trauma scenario)** | **0.720*** | -0.080 | 0.380 | 0.104 | 0.116 | 0.693 |
| **Q5 (obstructed intestinal tract scenario)** | 0.686 | 0.123 | 0.545 | 0.045 | 0.094 | 0.794 |
| **Q6** | 0.250 | 0.319 | 0.114 | 0.693 | -0.048 | 0.660 |
| **Q7** | 0.177 | 0.222 | 0.324 | 0.656 | -0.154 | 0.640 |
| **Q8** | 0.148 | 0.565 | 0.154 | 0.340 | 0.358 | 0.609 |
| **Q9** | 0.153 | **0.727*** | 0.087 | 0.342 | 0.052 | 0.679 |
| **Q10** | 0.064 | **0.838*** | -0.039 | 0.245 | -0.063 | 0.772 |
| **Q11** | 0.008 | **0.806*** | -0.049 | 0.121 | -0.185 | 0.701 |
| **Q12** | 0.272 | 0.640 | 0.197 | 0.045 | 0.252 | 0.588 |
| **Q (easy to use the game)** | 0.262 | 0.233 | 0.117 | **0.713*** | 0.226 | 0.696 |
| **Score for post-operative haemorrhage scenario** | -0.186 | 0.104 | -0.055 | 0.627 | 0.539 | 0.732 |
| **Score for brain trauma scenario** | -0.078 | -0.060 | 0.100 | 0.014 | **0.845*** | 0.734 |
| **Score for obstructed intestinal tract scenario** | 0.082 | 0.132 | 0.409 | 0.057 | 0.611 | 0.568 |
|  |  |  |  |  |  |  |
| **Explained variance** | 18.2% | 14.6% | 15.3% | 10.7% | 8.6% | 67.4% |

**b)** Factorial analysis (Principal Component Analysis, PCA) testing various aspects of the questionnaire (realism, educational content and impact on the player) (scores and questions in Tables 2 and 3).

* indicates loadings > 0.7 (i.e., the factor explains >49% of the item variance)

Communalities estimate the proportion of variance of an item explained by all factors.

|  | **Factor 1** | **Factor 2** | **Factor 3** | **Factor 4** | **Communalities**  **h²** |
| --- | --- | --- | --- | --- | --- |
| **Q1 (post-operative haemorrhage scenario)** | **0.734*** | 0.093 | 0.176 | 0.197 | 0.617 |
| **Q1 (brain trauma scenario)** | **0.763*** | 0.253 | 0.147 | 0.087 | 0.675 |
| **Q1 (obstructed intestinal tract scenario)** | **0.827*** | 0.134 | 0.040 | 0.014 | 0.704 |
| **Q2** | 0.103 | 0.428 | 0.489 | -0.029 | 0.434 |
| **Q3 (post-operative haemorrhage scenario)** | 0.144 | 0.141 | **0.824*** | 0.120 | 0.734 |
| **Q3 (brain trauma scenario)** | 0.149 | -0.004 | **0.795*** | 0.235 | 0.709 |
| **Q3 (obstructed intestinal tract scenario)** | 0.178 | 0.005 | **0.800*** | 0.086 | 0.679 |
| **Q4** | 0.649 | 0.118 | 0.031 | 0.285 | 0.517 |
| **Q5 (post-operative haemorrhage scenario)** | 0.575 | -0.014 | 0.576 | 0.246 | 0.723 |
| **Q5 (brain trauma scenario)** | **0.710*** | -0.069 | 0.401 | 0.136 | 0.688 |
| **Q5 (obstructed intestinal tract scenario)** | 0.658 | 0.125 | 0.575 | 0.099 | 0.789 |
| **Q6** | 0.214 | 0.288 | 0.073 | **0.751*** | 0.698 |
| **Q7** | 0.183 | 0.163 | 0.198 | **0.738*** | 0.644 |
| **Q8** | 0.064 | 0.588 | 0.270 | 0.363 | 0.555 |
| **Q9** | 0.122 | **0.734*** | 0.100 | 0.354 | 0.689 |
| **Q10** | 0.071 | **0.835*** | -0.066 | 0.232 | 0.760 |
| **Q11** | 0.060 | **0.790*** | -0.122 | 0.098 | 0.652 |
| **Q12** | 0.243 | 0.667 | 0.279 | 0.047 | 0.584 |
| **Q (easy to use the game)** | 0.169 | 0.243 | 0.160 | **0.756*** | 0.685 |
|  |  |  |  |  |  |
| **Explained variance** | 19.9% | 16.6% | 17.5% | 12.1% | 66.1% |

**c)** Factorial analysis (Principal Component Analysis, PCA) between the clinical reasoning process components (self-questionnaire) and errors made during the game (communication and situational awareness). Two students were excluded (one because data on the post-operative haemorrhage scenario were lacking and another because he/she did not answer the questionnaire).

* indicates loadings > 0.7 (i.e. the factor explains >49% of the item variance)

Communalities estimate the proportion of variance of an item explained by all factors.

*Global population*

|  | **Factor 1** | **Factor 2** | **Factor 3** | **Communalities**  **h²** |
| --- | --- | --- | --- | --- |
| **Situational awareness errors** | -0.012 | 0.010 | **-.948*** | 0.899 |
| **Communication errors** | 0.009 | **0.950*** | -0.012 | 0.903 |
| **Collect** | **0.762*** | 0.272 | 0.006 | 0.655 |
| **Process** | 0.665 | 0.054 | 0.187 | 0.480 |
| **Identify** | **0.785*** | -0.145 | 0.151 | 0.660 |
| **Establish goal** | **0.818*** | 0.057 | -0.181 | 0.705 |
| **Take action** | **0.700*** | -0.248 | -0.207 | 0.594 |
|  |  |  |  |  |
| **Explained variance** | 40.0% | 15.2% | 14.8% | 70.0% |

*Student nurse (S)* *group*

|  | **Factor 1** | **Factor 2** | **Communalities**  **h²** |
| --- | --- | --- | --- |
| **Situational awareness errors** | -0.058 | -0.447 | 0.203 |
| **Communication errors** | 0.487 | -0.309 | 0.333 |
| **Collect** | 0.287 | **0.735*** | 0.623 |
| **Process** | -0.137 | **0.834*** | 0.714 |
| **Identify** | 0.568 | 0.418 | 0.497 |
| **Establish goal** | **0.834*** | 0.247 | 0.757 |
| **Take action** | **0.773*** | 0.027 | 0.598 |
|  |  |  |  |
| **Explained variance** | 28.0% | 25.2% | 53.2% |

*Recently graduated (R) group*

|  | **Factor 1** | **Factor 2** | **Factor 3** | **Communalities**  **h²** |
| --- | --- | --- | --- | --- |
| **Situational awareness errors** | 0.071 | 0.025 | **0.963*** | 0.933 |
| **Communication errors** | -0.693 | 0.365 | -0.091 | 0.622 |
| **Collect** | 0.195 | **0.868*** | 0.122 | 0.806 |
| **Process** | 0.094 | **0.898*** | -0.099 | 0.825 |
| **Identify** | 0.666 | 0.333 | -0.315 | 0.654 |
| **Establish goal** | **0.807*** | 0.282 | 0.059 | 0.734 |
| **Take action** | **0.745*** | 0.181 | 0.118 | 0.602 |
|  |  |  |  |  |
| **Explained variance** | 31.2% | 27.4% | 15.4% | 74.0% |

*Expert nurse (E) group*

|  | **Factor 1** | **Factor 2** | **Factor 3** | **Communalities**  **h²** |
| --- | --- | --- | --- | --- |
| **Situational awareness errors** | 0.076 | -0.021 | **0.992*** | 0.990 |
| **Communication errors** | -0.042 | **-0.943*** | 0.017 | 0.891 |
| **Collect** | 0.750* | -0.417 | 0.036 | 0.738 |
| **Process** | **0.856*** | 0.213 | 0.128 | 0.794 |
| **Identify** | **0.899*** | 0.128 | -0.144 | 0.845 |
| **Establish goal** | **0.853*** | -0.256 | 0.081 | 0.800 |
| **Take action** | **0.816*** | 0.064 | 0.152 | 0.693 |
|  |  |  |  |  |
| **Explained variance** | 50.1% | 17.1% | 15.0% | 82.2% |
